# 0.1 mm ePTFE versus autologous pericardium for hand-sewn trileaflet valved conduit: a comparative study

**DOI:** 10.1007/s10047-019-01107-5

**Published:** 2019-06-01

**Authors:** Huifeng Zhang, Ming Ye, Gang Chen, Bing Jia

**Affiliations:** 1 Department of Cardiovascular Surgery, Children’s Hospital of Fudan University, Shanghai, 201102 China; 2 Cardiovascular Center, Children’s Hospital of Fudan University, 399 Wanyuan Road, Minhang District, Shanghai, China

**Keywords:** Hand-sewn, Trileaflet valved conduit, Conduit failure, Polytetrafluoroethylene

## Abstract

A hand-sewn trileaflet valved conduit is reportedly better than a bovine jugular graft. However, the comparative efficacy and safety between 0.1 mm ePTFE and autologous pericardium in this surgical procedure remained undetermined. This single-center cohort study included 46 patients aged 3–146 months who received implanted simplified hand-sewn trileaflet valved conduits: 31 patients (Group A) received 0.1 mm ePTFE valved conduits and 15 patients (Group B) received autologous pericardium valved conduits. Perioperative and follow-up outcomes up to 3 years after the surgeries were evaluated. No perioperative complications or early mortality were observed in either group, while one Group A patient aged 46 months died 6 months after surgery due to residual ventricular septal defect. No patients in Group A developed severe regurgitation or stenosis in valves of the conduits, but two moderate stenosis by echocardiography, and seven patients in group B were deemed to be conduit dysfunction (two stenosis, three stenosis plus regurgitation, and the remaining two regurgitation). No conduits failure was detected in group A, while two patients in group B (one for severe stenosis and the other one for severe regurgitation). After 6, 12, and 36 months, 95.2%, 88.9%, and 88.9% of Group A patients and 92.3%, 68.4%, and 42.7% of Group B patients were free from valved conduit dysfunction. After the same follow-up periods, all Group A patients had no conduit failure and 92.3%, 80.8%, and 80.8% of Group B patients were free from valved conduit failure. Within the 3-year follow-up period, 0.1 mm ePTFE novel simplified hand-sewn trileaflet valved conduits appear to be associated with a lower incidence of graft failure than autologous pericardium valved conduits.

## Introduction

Accumulating evidence from the previous studies indicates that hand-sewn valved conduits for surgical construction of right-ventricular outflow tract (RVOT) have good clinical efficacy and safety along with acceptable rates of graft failure [1–4]. Moreover, results from our recent study—including 22 new simplified hand-sewn trileaflet valved conduits and 54 bovine jugular vein (BJV) grafts that underwent surgical construction of RVOT—showed that the novel simplified hand-sewn trileaflet valved conduits with the assistance of two specially designed templates seem to be associated with lower incidences of perioperative complication, graft failure, and early phase mortality compared with the conventional BJV grafts [5].

Although the previous studies used various materials for conduit valves (mostly 0.1 mm expanded polytetrafluoroethylene [ePTFE] or autologous pericardium), the favored material for conduit valves remained undetermined. In a long-term (15 years) follow-up study, fresh autologous pericardial valved conduits showed excellent long-term results, with 92% and 76% of patients without reintervention 5 and 10 years after surgery [6]. Results of another study with a follow-up duration over 7 years showed that no functional failure of valves was detected in 48 patients with 0.1 mm ePTFE valved conduits [7].

This observation was further confirmed by the result of a recent single-center study, which also indicated a superior medium-term performance of ePTFE valved conduits for patients that underwent pulmonary valve replacement [8]. However, to the best of our knowledge, a few studies have compared the safety and efficacy between 0.1 mm ePTFE and autologous pericardium valved hand-sewn conduits for the surgical construction of RVOT.

Therefore, in this study, we aimed to report the single-center experience of our institution in applying simplified novel hand-sewn trileaflet valved conduits with 0.1 mm ePTFE or autologous pericardium for the surgical reconstruction of RVOT. We compared perioperative and subsequent follow-up outcomes of patients who received 0.1 mm ePTFE valved conduits against those who received autologous pericardium valved conduits.

## Materials and methods

### Patients

Forty-six patients who underwent simplified hand-sewn trileaflet valved conduits implantation from January 2013 to December 2017 in our institution were retrospectively analyzed; 31 patients (Group A) with 0.1 mm ePTFE valved conduits and 15 patients (Group B) with autologous pericardium valved conduits. The autologous pericardium valved conduits were mostly applied in the primary surgery to get big enough autologous pericardium, while 0.1 mm ePTFE valved conduits had no limitation. The median age of the patients was 54 months (range 3–146 months) and the median weight was 18 kg (5–42 kg). Legal guardians of all patients signed consent forms and the study protocol was approved by the institutional ethic committee prior to the commencement of the study.

### Suturing technique for hand-sewn valved conduit

**Fig. 1:**
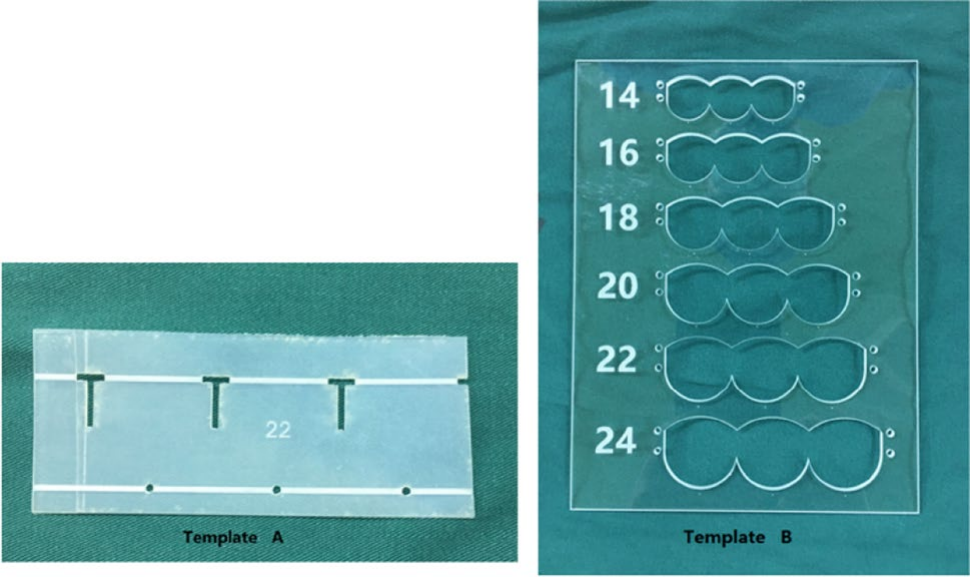
Representative images for Template A and Template B. Template A and Template B were designed to manufacture 14-, 16-, 18-, 20-, 22-, and 24-sized valved conduits. Template A was for marking “T” lines at every third of the circumference of the conduit; Template B was designed for tailoring membrane as three continuous semilunar shapes

**Fig. 2:**
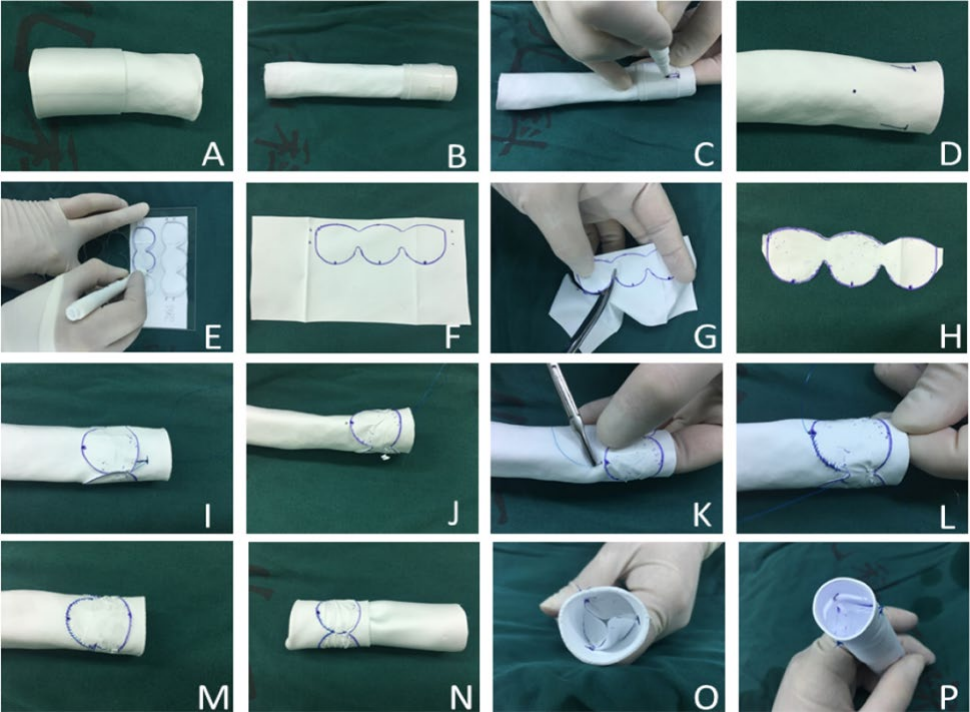
Schemes for the protocols of hand-sewn valved conduit. **a** Turning the Gore-Tex conduit inside out; **b, c, d** wrapping the conduit with template A to mark three “T” lines and dots; **e, f, g, h** 0.1 mm ePTFE membrane marked as three continuous semilunar shape with template B and then tailored; **i** the extra 2 mm of two ends of membrane overlapped and sutured to one “T” line with in-and-out vertical pledgeted suture; **j** Two conjunctions between both semilunar sheets stitched to the other two “T” lines with pledgeted 5–0 polypropylene; **k, l, m** The suture initiated two marked dots at the bottom of semilunar sheet to both sides until three semilunar sheets were completed; **n** The conduit was turned inside out again; **o** Every commissure was attached by one vertical mattress stitch. **p** Water testing showed that the valve was completely competent

Two templates (Fig. 1a, b) were used during the construction of valved conduits. A Gore-Tex® conduit (WL Gore and Associates Inc, Flagstaff, AZ) was tailored to the required length and turned inside out (Fig. 2a). Three “T” lines and points were marked at each third of the circumference, with template A (Fig. 1) wrapping the conduit (Fig. 20). A 0.1 mm ePTFE membrane (WL Gore and Associates Inc) or an autologous pericardium patch were tailored as three continuous semilunar shapes (Fig. 2e–h) with template B (Fig. 1). The conduit was wrapped with the tailored membrane located 5–10 mm (depending on conduit size) away from the distal conduit. The extra 2 mm of the two ends of membrane was overlapped and sutured to form one “T” line with in-and-out vertical continuous sutures (Fig. 2i, j) while two conjunctions of the semilunar sheet were stitched to the other two “T” lines with pledgeted 5–0 polypropylene. Then, the membrane was fixed to the conduit from the beginning of the bottom to the top of “U” sheet (Fig. 2k–m). After fixing of the membrane to the conduit, the conduit was turned inside out again (Fig. 2n). Every commissure was usually attached by one vertical mattress stitch (Fig. 2o).

### Surgical procedures

First, cardiac malformation was repaired under cardiac arrest during standard cardiopulmonary bypass. The minimal conduit size was calculated using standard tables estimated based on the weights of the patients. Hand-sewn trileaflet valved conduit was tailored into a slanting shape to increase the size of anastomosis to the pulmonary artery at its distal end. The right-ventricular outflow tract was reconstructed with the conduit implanted with end-to-end anastomosis with the pulmonary artery. The conduit valve was implanted as close to pulmonary artery bifurcation as possible to prevent potential regurgitation due to conduit twist caused by blood flow filling. The proximal end of the conduit was cut into a redundant anterior flap to construct the hood of the outflow tract and anastomosed with the right-ventricle incision. Conduit with inner diameter less than 14 mm was enlarged with the autologous pericardium or bovine pericardium (BalMedic Company, Beijing, China) to prevent proximal anastomosis stenosis.

Cefazolin (50 mg/kg) was administered intravenously before sternal incision and continued every 12 h for 3 days. Heparin was also administered (5–15 u/kg/h) intravenously for 4–5 h postoperatively, and warfarin was started as a replacement for heparin, so that the international normalized ratio (INR) reached 1.5–2.0 during the subsequent three months. Oral aspirin was then prescribed for 2 years.

### Demographic and perioperative information

Baseline characteristics—defined as age, gender, previous history of surgeries, and diagnosis of cardiac defects—were collected from each patient at admission. Perioperative information including cross-clamp time, cardiopulmonary bypass time, ventilation duration, duration of intensive-care unit stay, and volume of bleeding was also recorded. Active bleeding was diagnosed if the bleeding volume was more than 5 ml/h/kg within 3 h. Incidences of postoperative complications such as delayed sternal closure, low cardiac output syndrome, and complete AV block were also recorded.

### Conduit condition

Doppler echocardiography was applied to evaluate the severity of the conduit stenosis and regurgitation during the follow-up. Conduit insufficiency was graded as none or trivial, mild, moderate, and severe in accordance with the features of the jet flow measured by pulsed Doppler echocardiography as previously described [9]. The grade of the conduit stenosis was defined as mild or less if the peak velocity was < 3 m/s and the peak gradient was < 36 mmHg; moderate if the peak velocity was 3–4 m/s and the peak gradient was 36–64 mmHg; and severe if the peak velocity was greater than 4 m/s and the peak gradient was greater than 64 mmHg.

Conduit dysfunction was considered if moderate or greater stenosis (peak velocity > 3 m/s) was observed; moderate or greater insufficiency of the valves was confirmed by echocardiography [10]. Conduit failure was defined if surgical conduit replacement or transcatheter conduit dilatation was needed because of severe stenosis of the conduits or severer than moderate regurgitation of the valves [11].

### Endocarditis

Endocarditis was diagnosed via confirmation of newly developed conduit vegetation visualized on echocardiography or at least twice of positive results for the blood culture [12]. Treatment mainly included antibiotics for at least 6 weeks until negative results for blood culture were achieved. If conduit stenosis due to the endocarditis was hemodynamically significant, conduit replacement was recommended.

### Statistical analysis

Statistical analyses were performed using SPSS v16.0 software (SPSS, Inc., Chicago, IL). Continuous variables were presented as means ± standard deviations (SD) for normally distributed data or as medians and interquartile ranges otherwise. For subgroup analyses, the Student t test was used to compare the parametric variables, and the Mann–Whitney U test was used to compare the nonparametric variables.

One-way ANOVA was applied to analyze the different degree of the valve regurgitation and conduit stenosis between two groups. The incidences of conduit failure between two groups were analyzed using Kaplan–Meier analysis. A probability (*P*) value of < 0.05 was considered to be statistically significant.

## Results

### Patient characteristics

There were totally forty-six patients underwent hand-sewn trileaflet valved conduits implantation including thirty-one 0.1 ePTFE valved conduits as group A and fifteen autologous pericardium valved conduits as group B. The median age of all patients to get conduits implantation was 54 months (range 3–146 months) and the median weight was 18 kg (5–42 kg). Demographic characteristics and the primary diagnosis of the included patients are summarized in Table 1. There were no significant differences in the median ages, body weights, or oxygen saturations between the patients of the two groups. However, for patients who needed a second operation, 0.1 mm ePTFE valved conduits were applied dominantly compared with autologous pericardium valved conduits (20/31 versus 5/15; *P* = 0.047).

**Table 1:** Characteristics of the patients in each group

Characteristics	Group A (*n* = 31)	Group B (*n* = 15)	*P* value
Age, months, median (range)	54.0 (5–146)	37.0 (3–108)	0.30
Gender (male/female)	17/14	9/6	0.74
Weight, kg, median (range)	18.0 (6–34)	16.0 (5–42)	0.76
Oxygen saturation, median (%)	90.0 (60–98)	86.0 (60–99)	0.70
Previous operations	20 (64.5)	5 (33.3)	0.047
LVEF before surgery by ECHO (%)	68.3 ± 5.9	66.0 ± 8.2	0.29
Primary diagnosis			
PA/VSD	12 (38.7)	6 (40.0)	0.93
TGA complex	6 (19.4)	2 (13.3)	0.61
AS, AR	5 (16.1)	5 (33.3)	0.19
Truncus arteriosus	3 (9.7)	2 (13.3)	0.71
PR in repaired TOF	5 (16.1)	0 (0)	0.10
Surgical procedures			
Rastelli + VSD closure	7 (22.6)	7 (46.7)	0.01
Rastelli + fenestrated VSD closure	2 (6.5)	1 (6.7)	0.98
Ross ± Konno	5 (16.1)	5 (33.3)	0.19
Senning + switch/Rastelli	1 (3.2)	1 (6.7)	0.59
Nikaidoh	2 (6.5)	1 (6.7)	0.98
Conduit for PR in repaired TOF	5 (16.1)	0 (0)	0.10
Conduit replacement	9 (29.0)	0 (0)	0.03

Values are *n* (%) unless otherwise indicated; Data were presented as number (percentage)

*LVEF* left-ventricle ejection fraction, *VSD* ventricular septal defect, *PA/VSD* pulmonary atresia and ventricular septal defect, *TGA* transposition of the great arteries, *AS* aortic valve stenosis, *AR* aortic valve regurgitation, *TOF *tetralogy of Fallot, *PR* pulmonary regurgitation

### Surgical outcomes

Compared to autologous pericardium valved conduits, 0.1 mm ePTFE trileaflet valved conduits were primarily applied to replace the failed BJV grafts and for the patients with pulmonary regurgitation in repaired tetralogy of Fallot, because no enough autologous pericardium was available for valve-making in the patients for the second surgery (Table 1). No early mortality occurred in either group.

One patient aged 46 months with 0.1 mm ePTFE valved conduits died 6 months after surgery of congestive cardiac failure caused by the residual ventricular septal defect. No significant difference was observed between the two groups in incidence of bleeding, delayed sternal closure, low cardiac output symptom, or unrecovered complete atrioventricular block (Table 2).

**Table 2:** Surgical characteristics and follow-up outcomes for the patients in each group

Characteristics	Group A (*n* = 31)	Group B (*n* = 15)	*P* value
Aortic clamp time (min)	72.7 ± 39.2	93.5 ± 29.6	0.08
CPB time (min)	135.5 ± 49.1	147.1 ± 36.8	0.42
Conduit diameter, mm, media (range)	20 (14–22)	18 (14–22)	0.27
Ventilation, days, median (range)	2.5 (0.1–8)	3 (1–7)	0.19
ICU stay, days, median (range)	5 (1.4–15)	6 (3–17)	0.11
Early death	0 (0)	0 (0)	
DSC	3 (9.7)	1 (6.7)	0.73
bleeding	5 (16.1)	2 (13.3)	0.81
LCOS	0 (0)	1 (6.7)	0.15
Complete AV block	0 (0)	0 (0)	
Follow-up, months, median (range)	19 (2–52)	24 (3–63)	0.47
Late death	1 (3.2)	0 (0)	0.48
Endocarditis	1 (3.2)	0 (0)	0.48
LVEF by the last ECHO(%)	69.4 ± 3.8	68.5 ± 2.7	0.43
Valve regurgitation None or trivial Mild Moderate Severe	16 (51.6) 15 (48.4) 0 (0) 0 (0)	0 (0) 10 (66.7) 4 (26.7) 1 (6.7)	0.00
Peak velocity < 3 m/s 3 ≤ to < 4 m/s ≥ 4 m/s	29 (93.5) 2 (6.5) 0 (0)	11 (73.3) 3 (20.0) 1 (6.7)	0.00
Conduit dysfunction	2 (6.5)	7 (46.7)	0.001
Conduit failure	0 (0)	2 (13.3)	0.038
Re-intervention	0 (0)	0 (0)	

Values are *n* (%) unless otherwise indicated

*CPB* cardiopulmonary bypass, *DSC* delayed sternal closure, *ICU* intensive-care unit, *LCOS* low cardiac output syndrome, *LVEF* left-ventricle ejection fraction

### Post-operational outcome

The median follow-up of patients in Group A and B was 19 months (range 2–52 months) and 24 months (range 3–63 months), respectively (*P* = 0.47). The conduits’ valve function in group A remained competent with none or trivial-to-mild regurgitation, while four patients in group B suffered from moderate regurgitation and one patient of severe regurgitation (Table 2). For all but two patients in group A, the conduits’ peak velocity as evaluated by echography remained less than 3 m/s, while four patients in group B were with conduits’ peak velocity higher than 3 m/s (including a case of severe stenosis with peak velocity > 4 m/s that was scheduled for replacement) (Table 2). Therefore, two patients in group A (both due to moderate stenosis), and seven patients in group B (two for moderate stenosis, three for moderate-to-severe stenosis plus moderate regurgitation, and the remaining two for moderate-to-severe regurgitation) were deemed to be conduit dysfunction (Table 2).

**Fig. 3:**
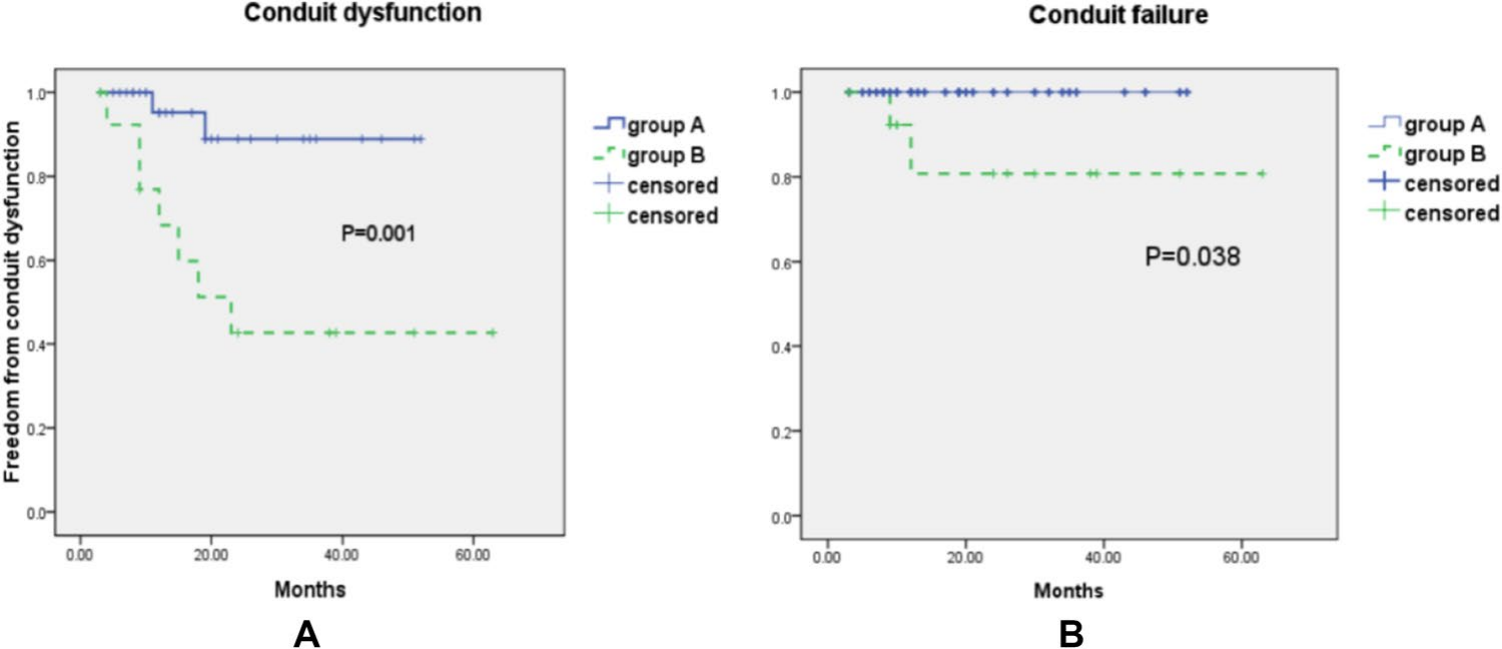
Follow-up analyses to determine the incidence of conduit dysfunction (**a**) and conduit failure (**b**) in the group A (solid line) and group B (dashed line) patients

Although no conduits’ failure was detected in group A, two patients in group B (one for severe stenosis and the other one for severe regurgitation) were considered as conduits failure. One patient with endocarditis was detected in group A (none in group B). This patient was diagnosed as endocarditis with *Candida albicans *infection and received emergent surgery with 0.1 mm ePTFE valved conduit replacement without sufficient antibiotic treatment due to the severe BJV graft obstruction. *Candida albicans* infection was re-detected in the patient after 3 months; this resolved after 2 months of antibiotic treatment without any sign of conduit failure. The proportions of patients that were free from valved conduits’ dysfunction in Group A within 6, 12, and 36 months were 95.2%, 88.9%, and 88.9%, while, of Group B, they were 92.3%, 68.4%, and 42.7% respectively (Fig. 3). The freedom from valved conduits’ failure within 6, 12, and 36 months was 100%, 100%, and 100% in Group A; 92.3%, 80.8%, 80.8%, respectively, in Group B (Fig. 3).

## Discussion

Results of our study revealed that the novel hand-sewn trileaflet valved conduits with 0.1 mm ePTFE or autologous pericardium were both associated with competent valve function with low incidences of conduit stenosis and perioperative complications. However, 0.1 mm ePTFE conduits seem to be more durable than autologous pericardium conduits. These results indicated that use of novel hand-sewn trileaflet 0.1 mm ePTFE valved conduits may improve the clinical outcome.

Considering that bovine jugular vein grafts were vulnerable of relatively high incidence of degeneration [13, 14], hand-sewn valved conduits were introduced, first by Schlichter [15], to reconstruct RVOT with autologous pericardium patch and ePTFE conduits. Hand-sewn valved conduits have been shown to be associated with lower incidence of conduit stenosis and better competent valve function compared with BJV grafts [16].

In recent years, a 0.1 mm ePTFE patch has been applied to the trileaflet valve, which was sewn inside the ePTFE conduit [17, 18]. Patients receiving new handmade 0.1 mm ePTFE conduits were found have a very low incidence of conduits failure and thrombosis, as reported by Ando [3] and Miyazaki [19]. In 2015, Shinkawa and associates [1] retrospectively analyzed 120 patients who underwent 0.1 mm ePTFE valved conduits. They showed that almost all patients receiving 0.1 mm ePTFE valved conduits were free from conduit reoperation (92.7% at 5 years). Results of our cohort showed that all valved conduits had valved competence and a low incidence of conduit stenosis except for two patients with autologous pericardium valved conduits, which were confirmed as conduit failure during mid-term follow-up. However, probably due to the limited number of cases in our study (46), we were unable to show that 0.1 mm ePTFE valved conduits were superior to autologous pericardium valved conduits. However, results of recent studies repeatedly showed favorable efficacies of 0.1 mm ePTFE valved conduits [20]. This advantage was considered due to the microporous structure of 0.1 mm ePTFE, which impeded cellular penetration, thereby making it resistant to calcification as well as minimal cellular and fibrous deposition.

On the contrary, autologous pericardium was prone to calcification as a bioprothetic material, which was shown relatively high mid-to-long-term dysfunction as the monocusp to reconstruct right-ventricular outflow [21]. The intermolecular collagen cross-linkages were suggested to play an important role in initiating autologous pericardium mineralization [22]. The blood flow dynamics in the pulmonary position were implied to be easy to induce the autologous pericardium degeneration [23]. Our early results also advocated the above findings; namely that 0.1 mm ePTFE was associated with a lower incidence of failure than autologous pericardium. Moreover, autologous pericardium for valve-making may be unavailable for some small children, especially in repeat operations. Calcification and contracture may be one of the underlying reasons why autologous pericardial conduits were associated with a little more regurgitation. Thus, 0.1 mm ePTFE material may be more appropriate for conduit valve-making.

Different methods for manufacturing valved conduits had been reported during the past 10 years, such as making bulging sinuses by Miyazaki [18] and clamping three ePTFE membrane with in and out vertical suture by Kim [24]. However, these methods remain time-consuming in calculating the width and length of the ePTFE patch with a ruler according to the different sized conduits. Moreover, bulging sinuses are problematic during surgery, since more time is required to manufacture conduits in advance in a very complicated process.

In our study, the application of the two templates while suturing the valved conduits ensured excellent function of all conduits even when sewn by different surgeons, and the operation could be completed within 30–40 min. Template a was used for positioning the suturing site, while template B was used for tailoring the ePTFE patch according to the chosen conduit size. These strategies may also be feasible, because they could reduce the manufacturing time and simplify the whole surgical process significantly.

The present study is limited by its retrospective and non-randomized design. Residual confounding factors were also possible in this study. Meanwhile, this study investigated a relatively small number of novel hand-sewn valved conduits with mid-term results. Obviously, larger studies are needed to strengthen these results.

In conclusion, patients with novel simplified hand-sewn trileaflet valved conduits made of 0.1 mm ePTFE appear to have lower incidences of perioperative complication and graft failure compared with patients with autologous pericardium valved conduits. These results should be confirmed in larger scale studies.
